# Metformin ameliorates the Phenotype Transition of Peritoneal Mesothelial Cells and Peritoneal Fibrosis via a modulation of Oxidative Stress

**DOI:** 10.1038/s41598-017-05836-6

**Published:** 2017-07-18

**Authors:** Hyun-Soo Shin, Jiyeon Ko, Dal-Ah Kim, Eun-Sun Ryu, Hye-Myung Ryu, Sun-Hee Park, Yong-Lim Kim, Eok-Soo Oh, Duk-Hee Kang

**Affiliations:** 1 Division of Nephrology, Department of Internal Medicine, Ewha Womans University School of Medicine, Ewha Medical Research Center, Seoul, Korea; 2 Division of Nephrology, Department of Internal Medicine, Kyung-Pook National University School of Medicine, Dae-gu, Korea; 3 Department of Life Sciences, Division of Life and Pharmaceutical Sciences, Ewha Womans University, Seoul, Korea

## Abstract

Phenotype transition of peritoneum is an early mechanism of peritoneal fibrosis. Metformin, 5′-adenosine monophosphate-activated protein kinase (AMPK) activator, has recently received a new attention due to its preventive effect on organ fibrosis and cancer metastasis by inhibiting epithelial-to-mesenchymal transition (EMT). We investigated the effect of metformin on EMT of human peritoneal mesothelial cells (HPMC) and animal model of peritoneal dialysis (PD). TGF-β1-induced EMT in HPMC was ameliorated by metformin. Metformin alleviated NAPDH oxidase- and mitochondria-mediated ROS production with an increase in superoxide dismutase (SOD) activity and SOD2 expression. Metformin inhibited the activation of Smad2/3 and MAPK, GSK-3β phosphorylation, nuclear translocalization of β-catenin and Snail in HPMCs. Effect of metformin on TGF-β1-induced EMT was ameliorated by either AMPK inhibitor or AMPK gene silencing. Another AMPK agonist, 5-amino-1-β-D-ribofuranosyl-imidazole-4-carboxamide partially blocked TGF-β1-induced EMT. In animal model of PD, intraperitoneal metformin decreased the peritoneal thickness and EMT with an increase in ratio of reduced to oxidized glutathione and the expression of SOD whereas it decreased the expression of nitrotyrosine and 8-hydroxy-2′-deoxyguanosine. Therefore, a modulation of AMPK in peritoneum can be a novel tool to prevent peritoneal fibrosis by providing a favorable oxidant/anti-oxidant milieu in peritoneal cavity and ameliorating phenotype transition of peritoneal mesothelial cells.

## Introduction

Maintenance of healthy peritoneum is one of the determinants for prognosis of patients on peritoneal dialysis (PD)^1, 2^. Accumulating evidences suggest epithelial-to-mesenchymal transition (EMT) as an early mechanism for peritoneal fibrosis^3–5^. Myofibroblasts produced in the process of EMT are known to provide an unfavorable environment for the preservation of peritoneal membrane via generation of pro-fibrotic cytokines and reactive oxygen species (ROS)^6, 7^. Despite recent controversies on EMT as a source of myofibroblast in the kidney and liver^8, 9^, previous data implicates EMT as a potential therapeutic target for preventing peritoneal fibrosis^3–5^.

Metformin is a semi-synthetic biguanide with two methyl groups attached to the nitrogen nucleus, and widely used to lower blood glucose in patients with type 2 diabetes mellitus. One of the mechanisms for an improvement of insulin sensitivity by metformin is an activation of 5′-adenosine monophosphate-activated protein kinase (AMPK)^10^. Interestingly, recent data have demonstrated the pleiotropic effects of metformin, which include an amelioration of organ fibrosis and a reduction of cancer incidence with an improvement of cancer patients’ survival^11–14^. To be more specific, metformin blocked transforming growth factor-β (TGF-β)-induced EMT of renal tubular cells and breast cancer cells^15, 16^, and also alleviated hepatic and cardiac fibrosis by blocking TGF-β signaling pathway via AMPK-dependent manner^17, 18^.

In this study, we investigated the effect of metformin and another AMPK agonist, 5-amino-1-β-D-ribofuranosyl-imidazole-4-carboxamide (AICAR) on EMT of peritoneal mesothelial cells with an exploration of an alteration in oxidative stress and AMPK activation. Effect of metformin on peritoneal fibrosis was also examined in animal model of peritoneal dialysis. Metformin decreased both NADPH oxidase (NOX)-mediated and mitochondrial ROS generation, and also alleviated EMT and peritoneal thickening via AMPK-independent and -dependent mechanism. This data suggest the potential therapeutic use of metformin or other AMPK agonists for the prevention and/or treatment peritoneal fibrosis.

## Results

### Metformin ameliorated TGF-β1-induced EMT of HMPCs

**Figure 1:**
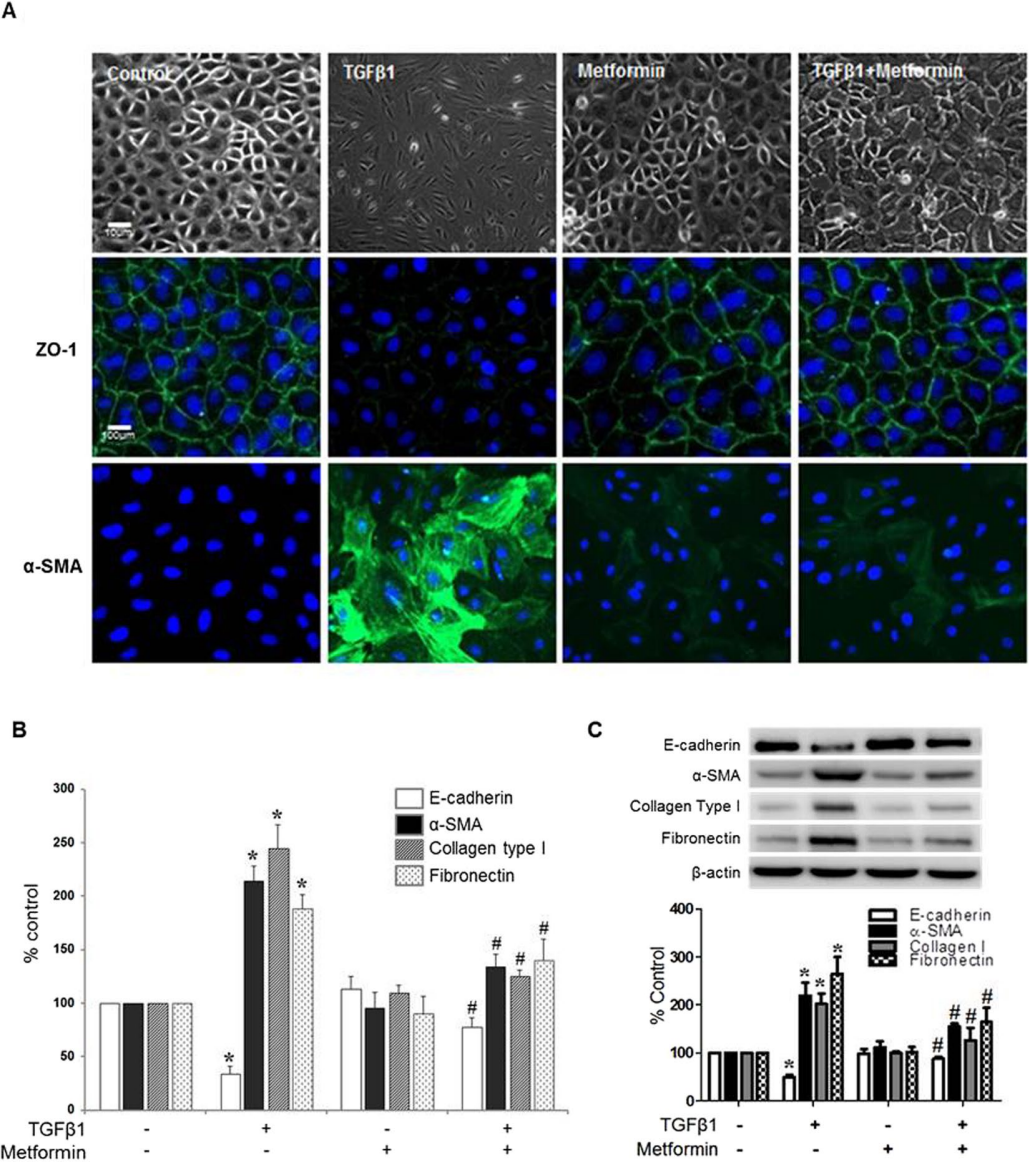
Effect of Metformin on TGF-β-induced EMT. TGF-β1 (1 ng/mL) induces the morphologic changes of HPMCs from a cuboidal, cobble-stone appearance to elongated, fibroblastoid morphology with decreased expression of ZO-1 and the acquisition of α-SMA expression in HPMCs. (**B**,**C**) Real-time PCR (**B**) and western blot analysis (**C**) reveal a decreased E-cadherin expression with an increase in α-SMA, collagen type I and fibronectin by TGF-β1. Metformin significantly ameliorates TGF-β1-induced EMT (**A**–**C**). Representative cell morphology and fluorescein immunocytochemistry for ZO-1 (green) and α-SMA (green) with nuclear DAPI staining (blue) at 48 hours of TGF-β1 with or without metformin (**A**). Quantitation bars for real-time PCR (**B**, n = 5) and representative western blots with quantitation bars (**C**, n = 6) are shown. *p < 0.05 vs. others, ^#^p < 0.05 vs. TGF-β1.

TGF-β1 (1 ng/mL) induced EMT shown as a morphologic change from a cuboidal shape to elongated spindle-shaped cells (Fig. 1A) with an altered expression of epithelial and mesenchymal cell markers (Fig. 1). Immunofluorescence staining, real time PCR and western blot analysis demonstrated a decreased expression of ZO-1 and E-cadherin, the markers of epithelial cells, with an increase in the expressions of α-SMA, collagen type I and fibronectin, the markers of mesenchymal cell, upon TGF-β1 stimulation. Metformin (10 μM) alleviated TGF-β1-induced alteration in cell morphology and the expression of epithelial and mesenchymal cell markers (Fig. 1).

### Metformin blocked TGF-β1-induced activation of Smad2/3, Erk and p38 MAPK

**Figure 2:**
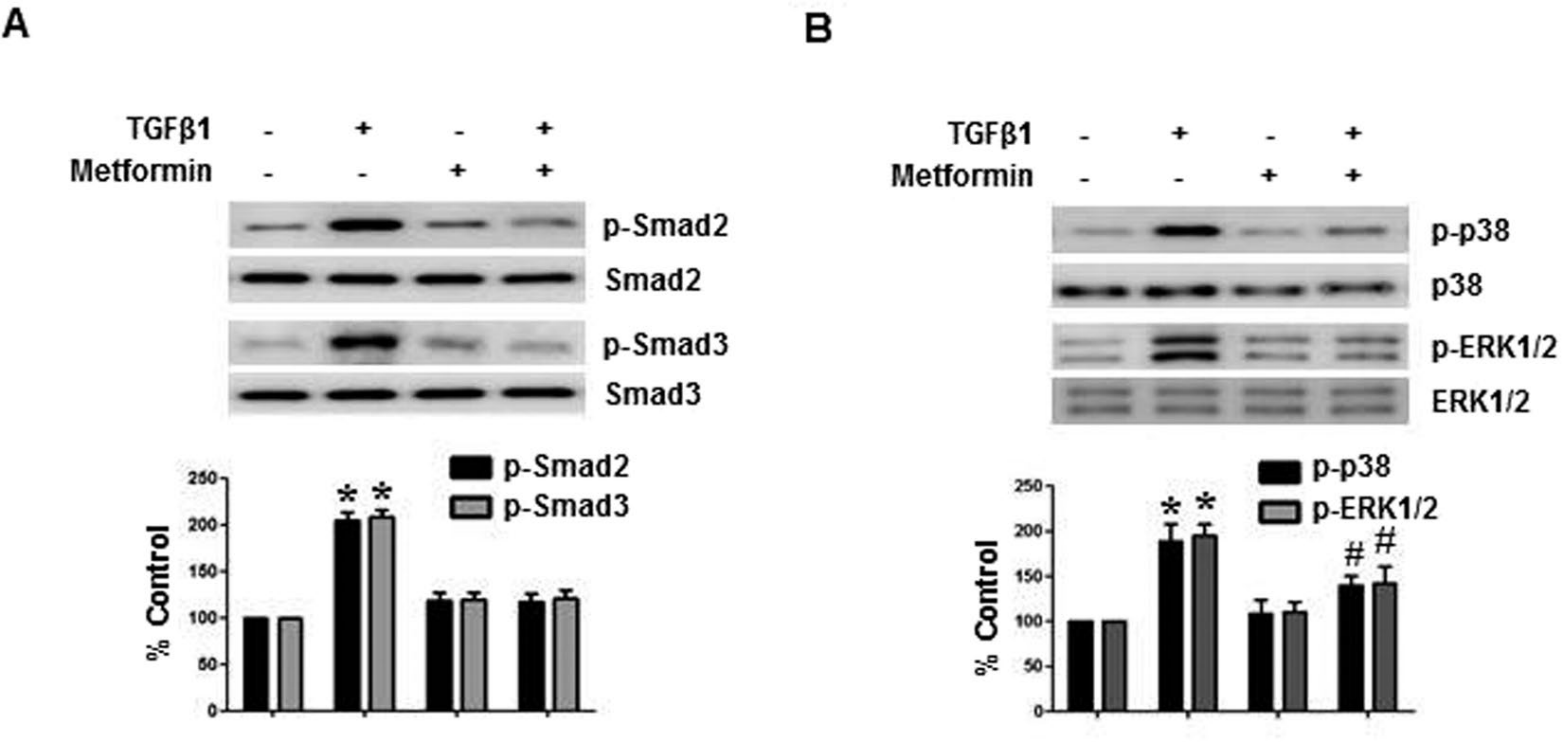
Effect of Metformin on TGF-β-induced Activation of Smad2/3 and MAPKinase Signaling. Metformin (1 µM) blocks TGF-β1-induced phosphorylation of Smad2/3 (**A**, 30 minutes), p38 and ERK1/2 MAPK (**B**, 3 hours). Representative western blots with quantitation bars (n = 6) are shown. *p < 0.05 vs. others, ^#^p < 0.05 vs. TGF-β1.

TGF-β1 induced the phosphorylation of Smad2/3 from 30 minutes (Supplemental Fig. 1A), and activated p38 and ERK1/2 MAPK pathways from 3 hours of stimulation (Supplemental Fig. 1B). Gene silencing of Smad2/3 by siRNA ameliorated TGF-β1-induced EMT (Supplemental Fig. 1C). Inhibition of p38 MAPK (SB203589, 10 μM) or ERK1/2 MAPK (PD98059, 10 μM) also alleviated EMT of HPMCs exposed to TGF-β1 (Supplementary Fig. 1C). Metformin treatment blocked an activation of Smad2/3, p38 and ERK MAPK in TGF-β1-exposed HPMCs (Fig. 2).

### Metformin inhibited TGF-β1-induced Snail Expression by blocking the phosphorylation of GSK-3β and nuclear translocation β-catenin

**Figure 3:**
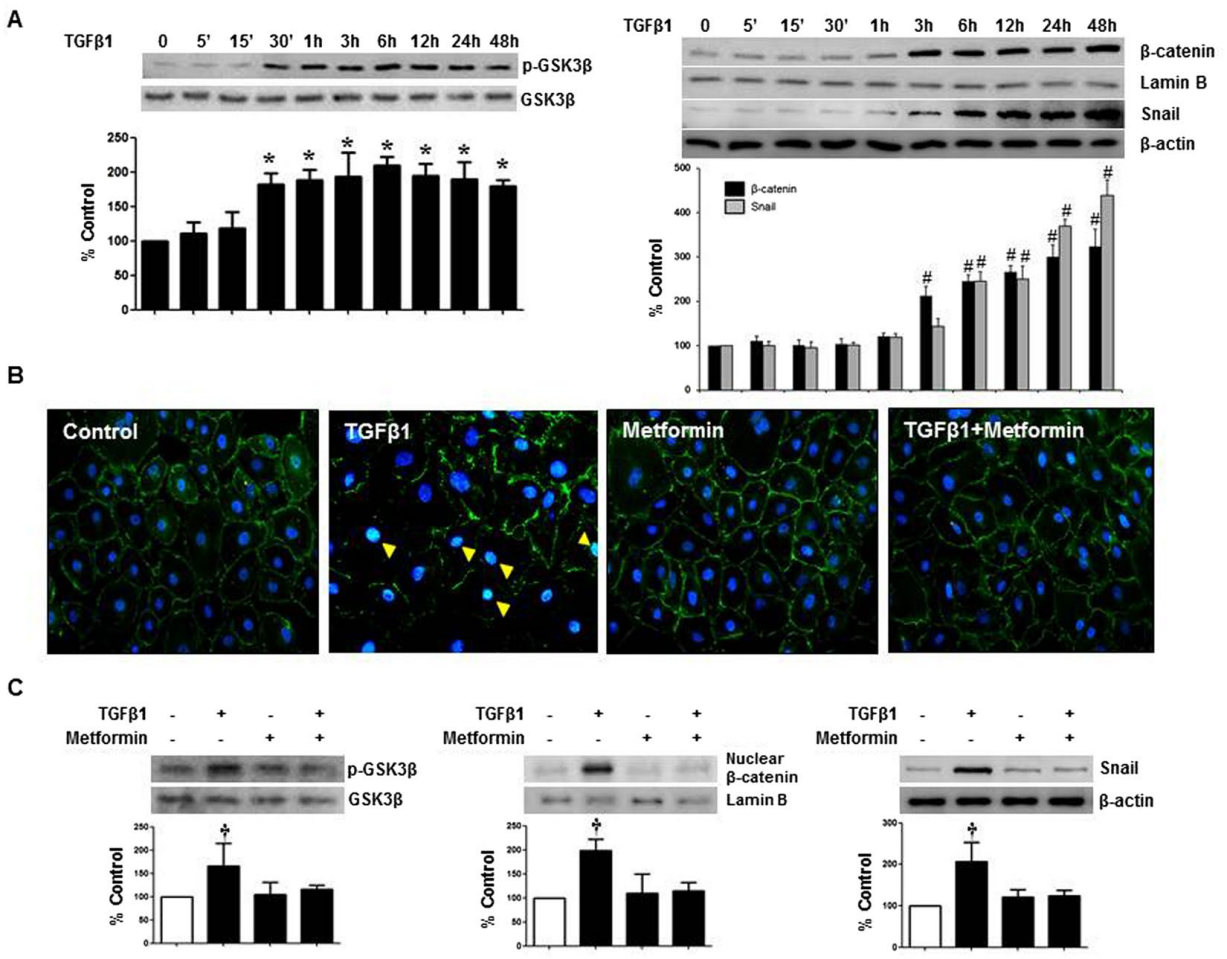
Effect of Metformin on TGF-β-induced Phosphorylation of GSK-3β, Nuclear Translocalization of β-catenin and Snail Expression. (**A**) TGF-β1 enhances the phosphorylation of GSK-3β, nuclear translocation of β-catenin and Snail expression. (**B**,**C**) Meformin (1 µM) significantly ameliorates TGF-β-induced activation of GSK-3β, nuclear translocalization of β-catenin and snail expression (**B**,**C**). Fluorescein immunocytochemistry of β-catenin (green) and DAPI (blue) reveals the translocation of β-catenin from the cytoplasmic membrane into the nucleus (cyan) upon TGF-β1 at 12 hours (**B**, yellow arrowheads). Representative western blots with quantitative analyses at 12 hours of TGF-β1 stimulation are shown (**A**,**C**). (n = 6). *p < 0.05 vs. time points of 0, 5, and 15 minutes, ^#^p < 0.05 vs. time points 0, 5, 15, 30, and 60 minutes, ^†^p < 0.05 vs. others.

To further understand the mechanisms responsible for TGF-β1-induced EMT in HPMCs, we investigated the activation of GSK-3β, nuclear translocation β-catenin, and Snail signaling pathway, which were known as a key mechanism of E-cadherin down-regulation and EMT^19–21^. TGF-β1 increased phosphorylation GSK-3β, nuclear translocation of β-catenin, followed by an increased expression of Snail in HPMCs (Fig. 3A and B). Metformin blocked the phosphorylation of GSK-3β, nuclear translocation of β-catenin, and Snail expression in HPMCs induced by TGF-β1 (Fig. 3B and C).

**Figure 4:**
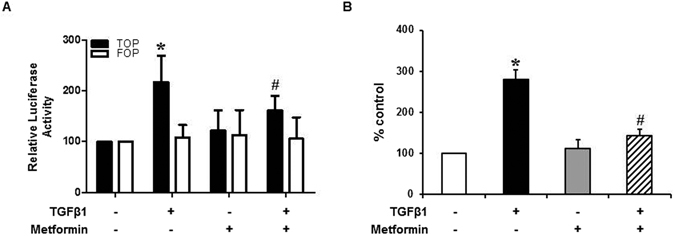
Effect of TGF-β and Metformin on β-catenin/TCF Transcriptional Activity. (**A**) Metformin exhibits a significant inhibitory effect against TOPflash reporter gene which was activated by TGF-β without affecting FOPflash activity (a mutant of the TCF binding site). Quantitation bar for relative Luciferase activity (n = 5) is shown. (**B**) Real-time PCR reveals the mRNA expression of matrix metalloproteinase (MMP)-7 is up-regulated by TGF-β1 (2.8-fold increase, 24 hours), which is ameliorated by metformin (N = 6). *p < 0.05 vs. others, ^#^p < 0.05 vs. TGF-β1.

To investigate the role of β-catenin/T-cell factor (TCF) signaling in TGF-β1-mediated EMT, TOP/FOPflash assay was used. TGF-β1 induced TOPflash reporter activity by ~2.2-fold but had no effect on FOPflash activity (a mutant of the TCF binding site) (Fig. 4A). Metformin ameliorated TGF-β1-induced activation of TOPflash activity, suggesting that the inhibitory activity by metformin on TGF-β1-mediated EMT could be dependent on β-catenin/TCF signaling. In addition, the mRNA expression of matrix metalloproteinase (MMP)-7, a representative down-stream target gene of β-catenin/TCF signaling in HPMCs, was up-regulated by TGF-β1 (2.8-fold increase, 24 hours, N = 6), which was blocked by metformin (Fig. 4B).

### Metformin blocked TGF-β1-induced ROS Production by NOX activation and Mitochondrial Dysfunction

**Figure 5:**
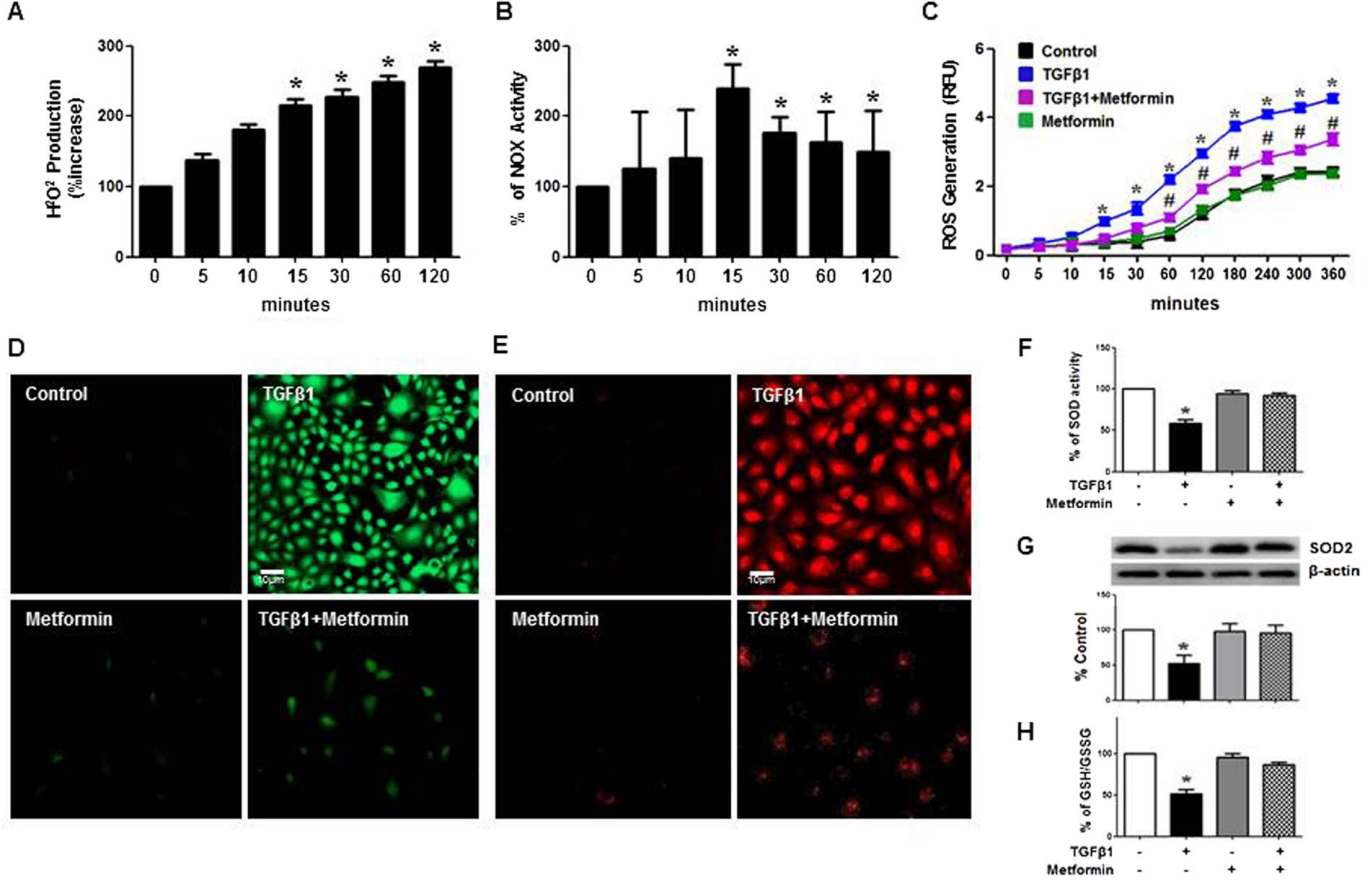
Effect of Metformin on TGF-β-induced Oxidative Stress. TGF-β1 increases H_2_O_2_ generation (**A**) with an enhanced activity of NOX (**B**) from 15 minutes. Metformin blocks ROS generation (**C**) assessed by DCF-DA staining (green) at 30 minutes (**D**) and Mito-SOX staining (red) at 6 hours (**E**). (n = 6). TGF-β1 decreases the activity of superoxide dismutase (SOD) (**F**, 6 hours) and the expression of SOD2 (**G**, 6 hours) and a release of reduced glutathione (GSH/GSSG) (**H**, 6 hours) in HPMCs, which was restored by metformin. Representative western blots with quantitation bar (**G**) (n = 6) are shown. *p < 0.05 vs. time points of 0, 5 and 10 minutes, ^#^p < 0.05 vs. control, TGF-β1 or metformin, ^†^p < 0.05 vs. others.

One of the earliest changes in TGF-β1-exposed HPMCs was an increased ROS production with an enhanced NOX activity, which was found in 15 minutes of TGF-β1 stimulation (Fig. 5A and B). Interestingly, metformin ameliorated ROS production in TGF-β1-exposed HPMCs (Fig. 5C and D). TGF-β1 also increased mitochondrial ROS production shown as an increased Mito-Sox staining in 6 hours of TGF-β1 stimulation in HPMCs, which was blocked by metformin (Fig. 5E).

### Metformin increased Anti-oxidant Activities in TGF-β1-exposed HPMCs

In addition to an amelioration of TGF-β1-induced oxidative stress in HPMC, metformin reversed a decrease in anti-oxidant activity by TGF-β1. Metformin increased an activity of superoxide dismutase (SOD) and the expression of manganese-dependent superoxide dismutase, which is also known as SOD2, with a release of reduced glutathione (GSH/GSSG) in HPMCs exposed to TGF-β1 (Fig. 5F–H).

### AMPK agonist also ameliorated TGF-β1-induced EMT

To investigate whether the effect of metformin on peritoneal EMT was mediated by AMPK activation, we examined the effect of another AMPK agonist, 5-amino-1-β-D-ribofuranosyl-imidazole-4-carboxamide (AICAR, 10 μM) on TGF-β1-induced EMT.

Interestingly, there was a timely differential activation of AMPK by metformin and AICAR. AICAR activated AMPK of HPMCs from 15 minutes whereas the effect of metformin on AMPK phosphorylation was observed from 24 hours (Supplementary Fig. 2), which was delayed compared to AICAR.

**Figure 6:**
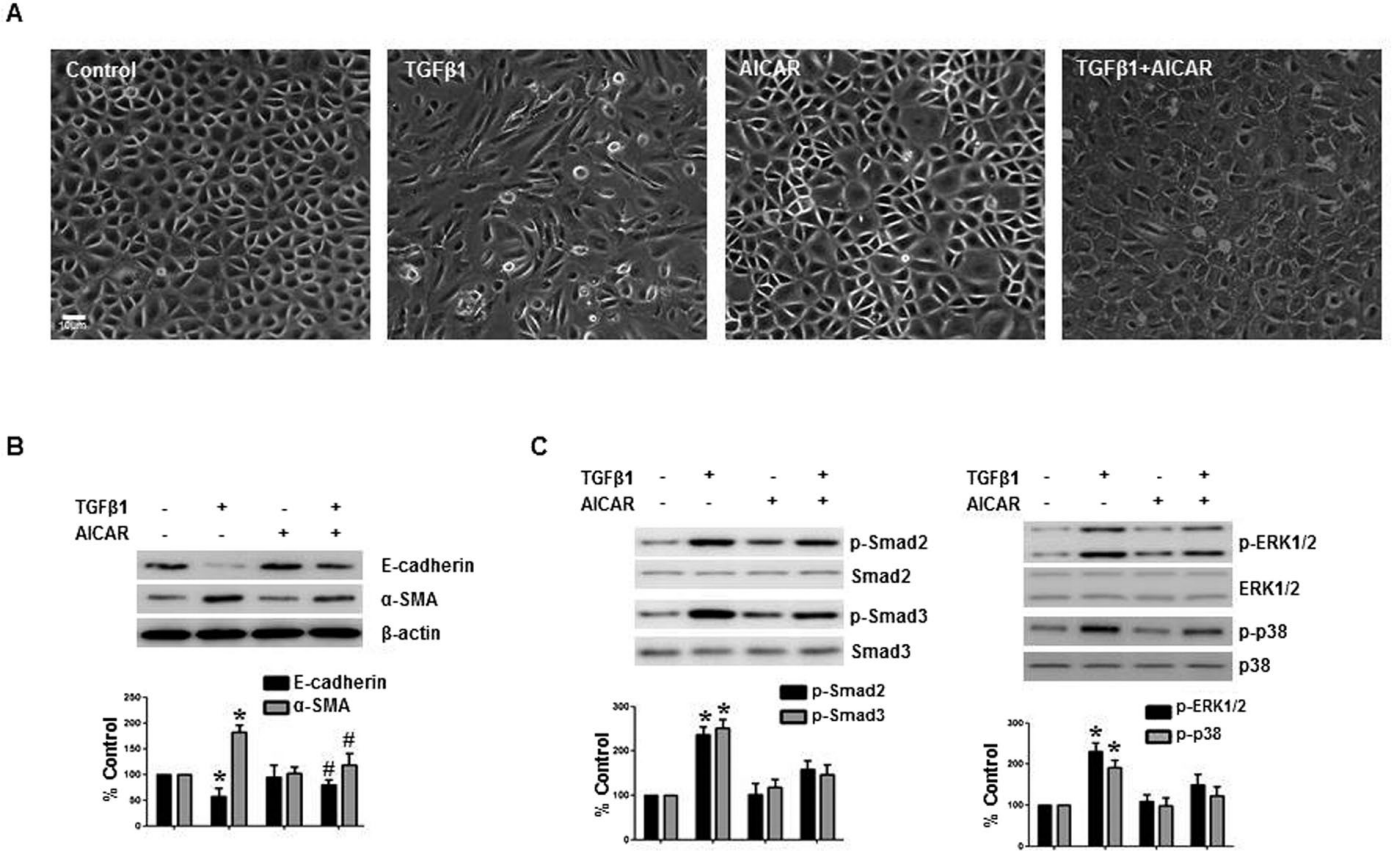
Effect of AMPK agonist, AICAR, on TGF-β-induced EMT and Alteration of Intracellular Signaling Pathways. AICAR (5-amino-1-β-D-ribofuranosyl-imidazole-4-carboxamide, 10 μM) alleviates TGF-β1-induced changes in cell morphology (**A**) and the expression of E-cadherin and α-SMA (**B**). AICAR does not block an activation of Smad2/3 pathway (30 minutes) and MAPK phosphorylation (3 hours) (**C**). Representative cell morphology (**A**) and western blotting with quantitation graph are shown (**B**–**D**). N = 6. *p < 0.05 vs. others, ^#^p < 0.05 vs. TGF-β1.

**Figure 7:**
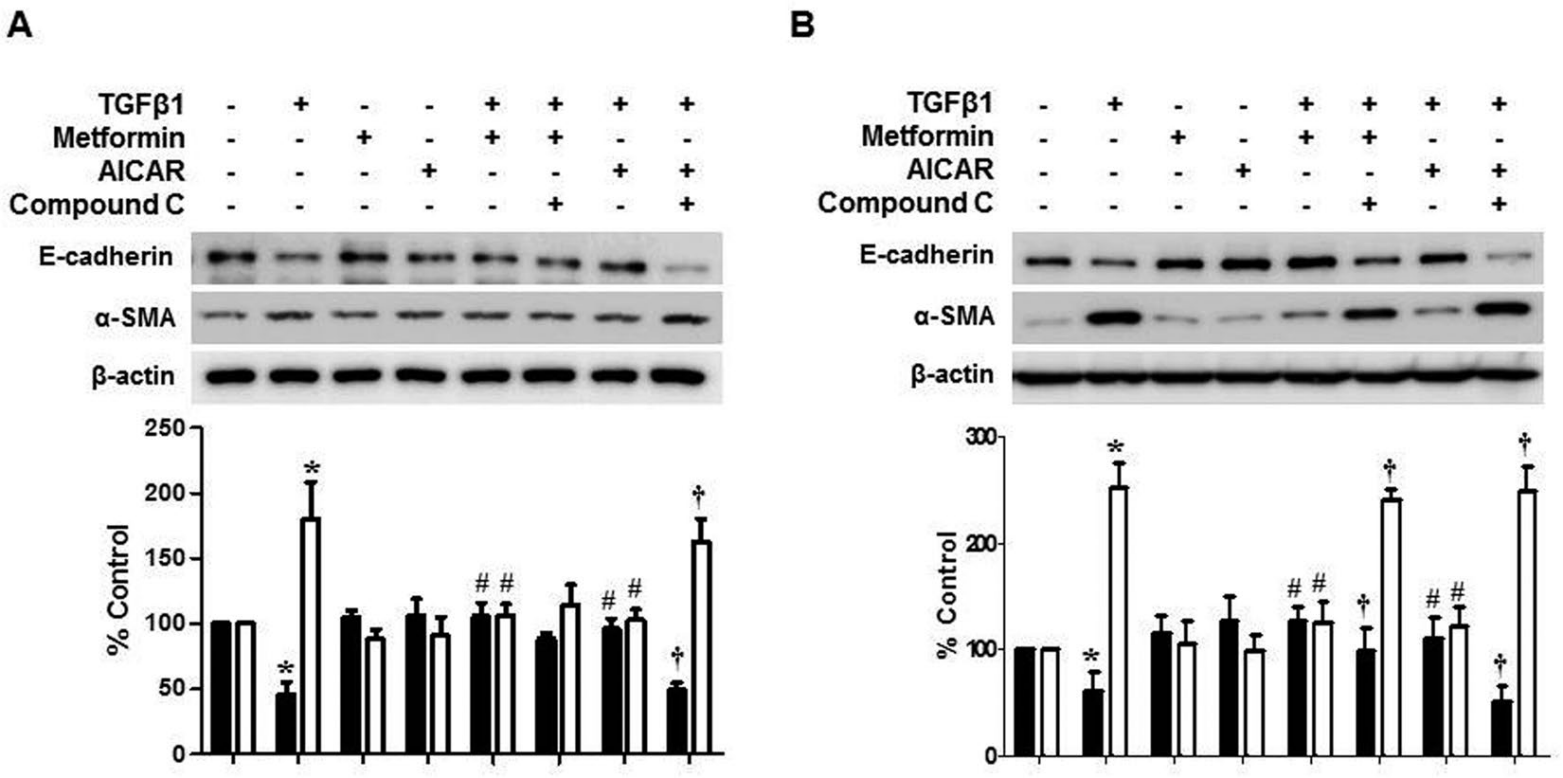
Differential Effect of Compound C on Metformin- and AICAR-induced Amelioration of EMT. Compound C, AMPK inhibitor, dose not reverse the protective effect of metformin on TGF-β1-induced EMT at 12 hours (**A**), but partially inhibits the effect of metformin at 48 hours of TGF-β1 stimulation (**B**). Effect of AICAR on TGF-β1-induced EMT is blocked by compound C at 12 (**A**) and 48 hours (**B**). N = 4. *p < 0.05 vs. others, ^#^p < 0.05 vs. TGF-β1, ^†^p < 0.05 vs. TGF-β1+ AICAR or metformin.

AICAR also alleviated TGF-β1-induced EMT (Fig. 6A,B), which was reversed by co-treatment with an AMPK inhibitor, compound C (Fig. 7). Interestingly, AICAR treatment did not induce a significant down-regulation of Smad2/3 and MAPK phosphorylation in contrast to metformin (Fig. 6C). Compound C (20 μg/mL) did not affect the effect of metformin on EMT at 12 hours, however partially reversed the effect of metformin on TGF-β1-induced EMT at 48 hours (Fig. 7) attributable to a delayed activation of AMPK by metformin (Supplementary Fig. 2).

**Figure 8:**
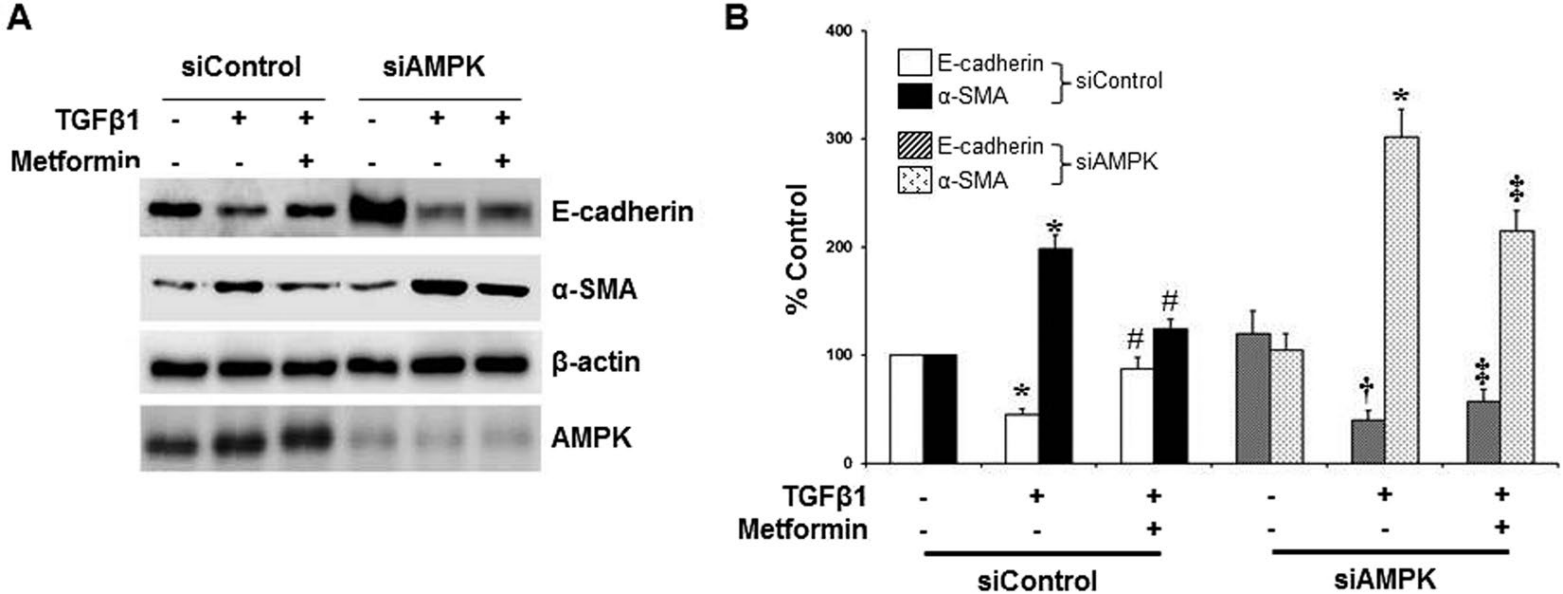
Effect of AMPK Gene Silencing on Amelioration of EMT by Metformin in HPMCs. AMPK gene silencing by siRNA does not affect the constitutional expression of E-cadherin and α-SMA. siAMPK alleviates the effect of metformin to block or ameliorate TGF-β1-induced changes in the expression of E-cadherin and α-SMA in HPMCs. Representative western blots (**A**) with quantitative bars (**B**) at 24 hours of TGF-β are shown. (n = 4). *p < 0.05 vs. others at each siRNA condition, ^#^p < 0.05 vs. TGF-β at siControl, ^†^p < 0.05 vs. E-cadherin of control at siAMPK, ^‡^p < 0.05 vs. E-cadherin or α-SMA of TGF-β+ metformin at siControl.

Gene silencing of AMPK by siRNA also blocked or alleviated the effect of metformin on TGF-β1-mediated EMT (Fig. 8).

### Metformin ameliorated EMT and Peritoneal Thickening in Animal Model of Peritoneal Dialysis

**Figure 9:**
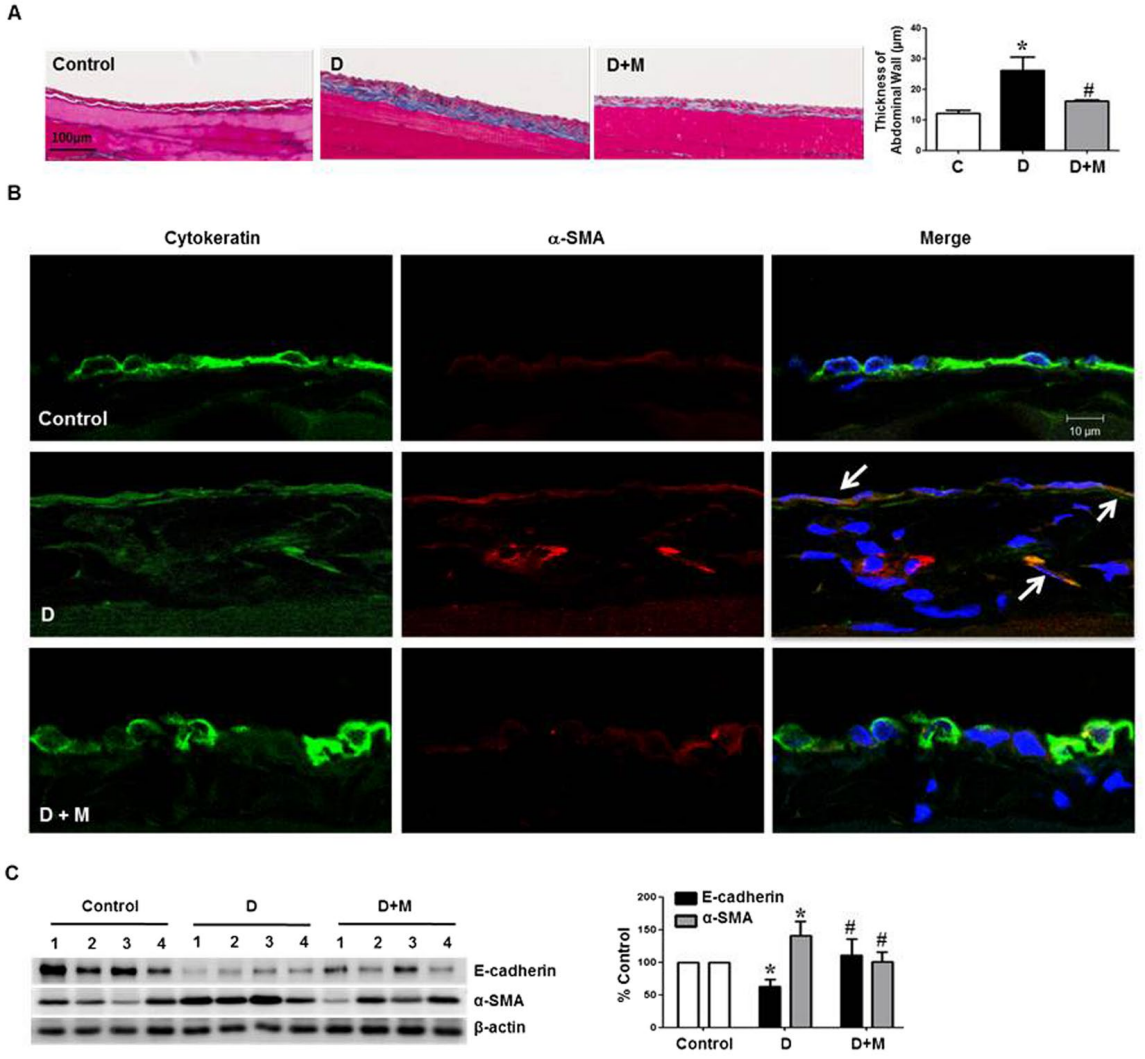
Effect of Metformin on Peritoneal Fibrosis and EMT in Animal Model of Peritoneal Dialysis. (**A**) Masson’s Trichrome-stained tissue of parietal peritoneum shows that peritoneal thickness in dialysis group (D) is significantly higher than those in control group, which is decreased in group D + M. Representative histology of parietal peritoneum is shown with quantitation bar of peritoneal thickness. Magnification ×200. Scale bar 100 μm. *p < 0.05 vs. others, ^#^p < 0.05 vs. group D. (**B**) Representative fluorescent confocal image of peritoneum shows a decreased cytokeratin (green) and increased α-SMA staining (red) in mesothelial lining with an appearance of cytokeratin (+)/α-SMA (+) cells in mesothelial and submesothelial zone (arrows). Rats in group D + M demonstrates a restoration of cytokeratin (+) mesothelial cells with a decrease in cytokeratin (+)/α-SMA (+) cells in mesothelial and submesothelial area. Magnification ×640. Scale bar 10 μm. (**C**) Western blot analysis with quantitation bar shows the changes of E-cadherin and α-SMA in group D are ameliorated by metformin treatment. *p < 0.05 vs. others, ^#^p < 0.05 vs. group D.

In 8 weeks of peritoneal dialysis, mean thickness of parietal peritoneum in PD group (26.2 ± 4.3 μm) was significantly higher compared with control (12.1 ± 1.0 μm). Intraperitoneal metformin injection (50 mg/kg/day) resulted in a decrease in peritoneal thickness (16.2 ± 0.3 μm) compared to dialysis group (group D) (Fig. 9A). Increased peritoneal thickness in group D was associated with an evidence of EMT shown as a decreased cytokeratin with an increased α-SMA staining in mesothelial lining and an appearance of cytokeratin (+)/α-SMA (+) cells in submesothelial zone (Fig. 9B). Rats on dialysis and metformin (group D + M) demonstrated a decrease in cytokeratin (+)/α-SMA (+) cells in mesothelial and submesothelial area, suggesting that metformin protected peritoneum from EMT. Western blot analysis also showed EMT in group D, which was alleviated by metformin treatment (Fig. 9C).

### Metformin restored Anti-oxidant/Oxidant Balance in Peritoneum

**Figure 10:**
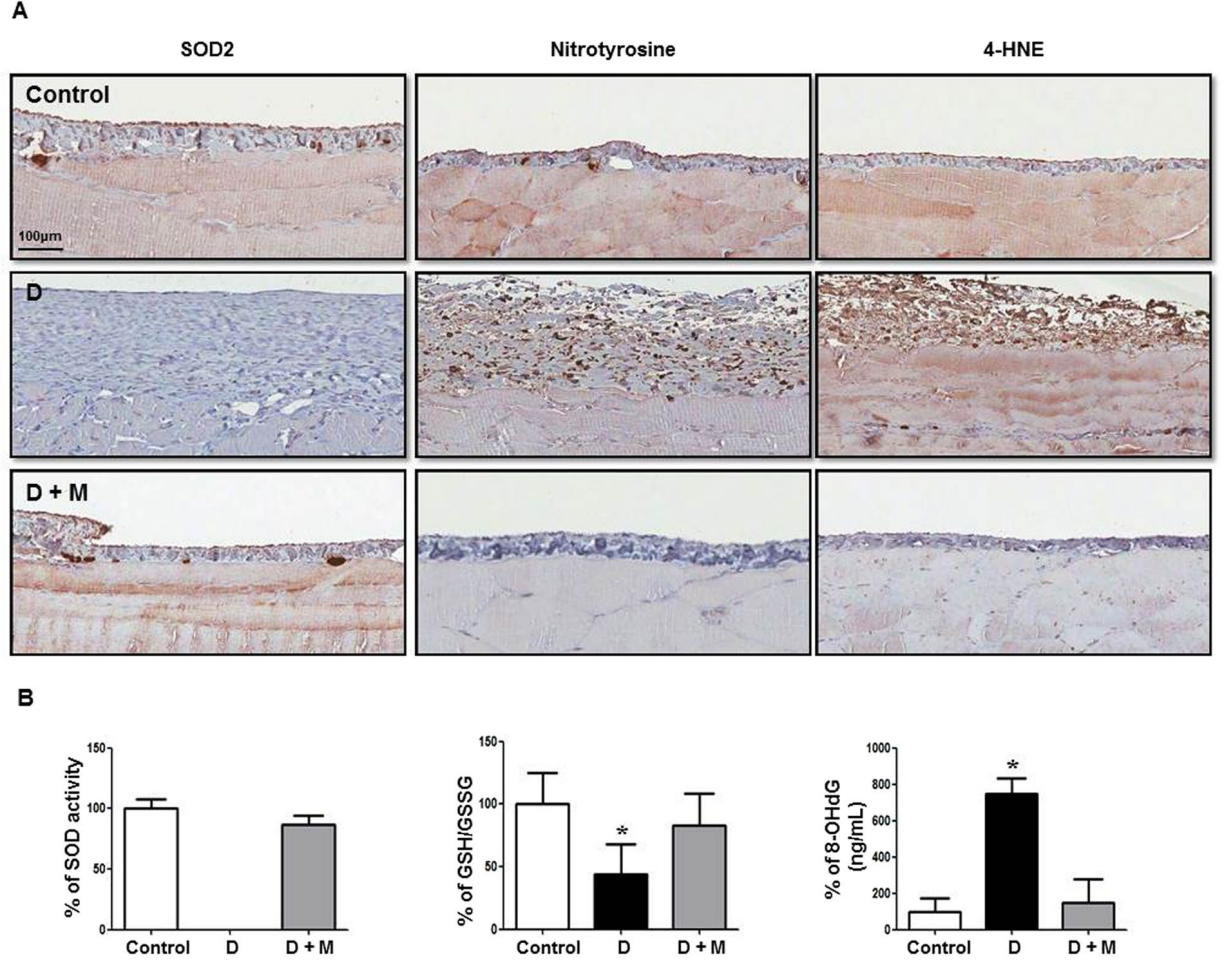
Effect of Metformin on Intraperitoneal Oxidative Stress in Animal Model of Peritoneal Dialysis. (**A**) In dialysis group (D), a decrease in superoxide dismutase 2 (SOD2) with an increased staining of nitrotyrosine (NT) and 4-hydroxynonenal (4-HNE) in peritoneal membrane is observed. Rats in group D + M demonstrates an alleviation of altered expression of SOD2, NT and 4-HNE. (**B**) SOD activity and GSH/GSSG in peritoneal effluent are significantly decreased in group D with an increase in 8-OH-dG, which are ameliorated in group D + M. Representative immunohistochemical staining of parietal peritoneum is shown (**A**) Magnification ×200. N = 4. *p < 0.05 vs. others.

Eight-week-peritoneal dialysis in rats resulted in a decrease in SOD2 with an increased oxidative stress shown as an intense staining of nitrotyrosine (NT) and 4-hydroxynonenal (4-HNE) in peritoneal membrane (Fig. 10A). Metformin treatment alleviated an altered expression of SOD2, NT and 4-HNE in rats on PD.

In peritoneal effluents, the levels of anti-oxidants such as SOD and GSH/GSSG were significantly decreased in group D with an increase in a marker of oxidative stress, 8-OH-dG (Fig. 10B). Particularly, SOD activity in peritoneal effluent was not detected in all 4 rats of group D. Metformin significantly restored the change in the markers of anti-oxidant/oxidative stress in peritoneal effluent (Fig. 10).

## Discussion

In this study, we demonstrate that (i) metformin ameliorates TGF-β1-induced EMT of peritoneal mesothelial cells and animal model of PD; (ii) the beneficial effect of metformin on EMT and peritoneal fibrosis is attributed to an inhibition of ROS generation, Smad2/3, ERK and p38 MAPKinase activation; (iii) the effect of metformin is independent of AMPK activation at early time points, however can be mediated by AMPK at later time points; (iv) metformin also protects the peritoneum from EMT and fibrosis by reinforcing local anti-oxidant activity. This is the first study to demonstrate the effect of metformin and AMPK agonist on peritoneal EMT both in *in-vitro* and *in-vivo* experiment.

The most important finding of this study is a validation of antifibrotic effect of metformin in peritoneal mesothelium. EMT is one of the earliest mechanisms of peritoneal fibrosis^4, 5, 22^. Mesothelial cells are known to undergo EMT to become matrix-producing myofibroblasts under pathologic conditions and participate in the pathogenesis of peritoneal fibrosis^4, 5, 7, 23–25^. Therefore, pharmacological prevention and/or reversal of EMT may serve as one of the possible therapeutic approaches to peritoneal fibrosis. In this study, TGF-β1 induced EMT with an activation of Smad2/3 and MAPKinase, followed by phosphorylation of GSK-3β and nuclear translocalization of β-catenin. It was already known that inactivation of GSK-3β by phosphorylation at threonine residue resulted in a mobilization of β-catenin into nucleus and an enhanced expression of transcription factors such as snail to down-regulate E-cadherin expression^26^. We confirmed a role of TGF-β1-induced activation of β-catenin/TCF signaling pathway assessed by TOPflash assay, nuclear staining and quantitation of β-catenin and real-time PCR of MMP-7 in EMT of HPMCs, which was blocked by metformin. Earlier studies have revealed that metformin inhibits EMT of breast cancer cells, renal tubular cells, and lung epithelial cells^11, 15, 27, 28^. A modulation of EMT by metformin in cancer cells is proposed as a mechanism explaining a reduced risk of developing cancer^29–31^. However, there have been no reports on the effect in peritoneal mesothelial cells, and this study is the first demonstrating the beneficial effect of metformin on peritoneal EMT and fibrosis.

The earliest phenomenon observed in TGF-β1-exposed peritoneal mesothelial cells was an activation of NOX which induced ROS generation. In this study, we confirmed not only membranous NOX but mitochondria also contributed to an accumulation and release of ROS in HPMCs. Mitochondrial ROS generation was observed at later time points, in 6 hours of TGF-β1 stimulation compared to an early activation of membranous NOX at 15 minutes. Oxidative stress is known as a major mechanism of high glucose or TGF-β1-induced EMT of peritoneal mesothelial cells^32^. Consistent to the result of previous report^24^, we observed anti-oxidants, including N-acetyl cysteine (NAC, ROS scavenger), apocynin (NOX inhibitor), mitoQ (an inhibitor of mitochondrial electron transfer chain subunit I) alleviated TGF-β1-induced EMT of HPMCs (Supplementary Fig. 3). Interestingly, metformin decreased H_2_O_2_ generation by inhibiting both NOX activation and mitochondrial ROS production. The primary target of metformin in intact cells is the mitochondrion, where it inhibits respiratory chain complex I. Metformin was also reported to reduce mitochondrial damage expressed as an increased mitochondrial DNA copy number, cytochrome c release, caspase 3/9 activation, and mitochondrial ROS production^33–36^. In addition to an amelioration of oxidative stress, metformin also enhanced anti-oxidant activity and the expression of SOD2 in mesothelial cells, which was demonstrated for the first time in this study (Fig. 5). SOD is an important antioxidant enzyme present in nearly all living cells. SOD2, one of the most abundant SODs in HPMCs in our preliminary experiment, was known to be located in mitochondria, and is reported to play a pivotal role in cardioprotection in ischemia-reperfusion injury to myocardium^37^. Our study also confirmed a favorable effect of metformin on oxidative stress in *in-vivo* study. Peritoneal dialysis for 8 weeks resulted in an increased accumulation of nitrotyrosine and 4-HNE in peritoneal membrane, which was alleviated by metformin (Fig. 10). Peritoneal dialysis also reduced GSH/GSSG, SOD activity with an increased 8-hydroxy deoxyguanosine in peritoneal dialysate. Particularly, SOD activity in peritoneal effluent was almost not detected in rats on peritoneal dialysis in this study. Recent paper demonstrated metformin blocked TGF-β, angiotensin II and high glucose-induced EMT of renal tubular cells via an upregulation of heme oxygenase and thrioredoxin^15^. Taken together, metformin seems to provide a favorable oxidant/anti-oxidant microenvironment in peritoneal cavity by restoration of oxidative stress/antioxidant balance.

Metformin is an agonist of AMPK. A decrease in ATP production by metformin activates the energy sensor AMPK^38^. In addition to its traditional role as a modulator of cellular and whole body energy homeostasis, AMPK is recently shown to be involved in an amelioration of inflammation, angiogenesis, and organ fibrosis^39–41^. In this study, we confirmed a constitutive expression of AMPK in HPCMS. Metformin activated AMPK in HPMCs from 24 hours, which was delayed compared to another AMPD agonist, AICAR which activated AMPK in 15 minutes. Therefore, the effects of metformin on ROS generation (from 15 minutes), the activation of intracellular signal pathways (from 30 minutes), and a down-regulation of E-cadherin (from 12 hours) seem to be independent of AMPK activation. Considering both the protective effect of AICAR on TGF-β1-induced EMT and the partial reversal of beneficial effect of metformin by AMPK inhibitor, compound C or AMPK gene silencing, AMPK is thought to play a role in modulation of peritoneal EMT and metformin alleviates peritoneal EMT via both AMPK-dependent (late) and independent (early) pathways.

Despite the beneficial effects of metformin not related to glycemic control, the prescription of metformin has been limited by a concern for the development of metabolic acidosis^42^. Metformin is eliminated unchanged by the kidney, and expected to accumulate in patients with reduced renal function. However, the most recent Cochrane review found no evidence of an increased incidence of lactic acidosis with metformin based on the data including 347 comparative trials and cohort studies including the patients with impaired renal function^43^. Recent follow-up study in peritoneal dialysis patients also showed no case of lactic acidosis with metformin (0.5–1.0 g/day) for 4 weeks in 37 automated PD patients^44^. Based on this observation, the authors suggested that metformin could be used in PD patients with caution. It is currently not clear whether metformin per se is a cause of lactic acidosis in renal failure patients because acidosis in these patients is almost always associated with other causes of metabolic acidosis including sepsis, hypovolemia, ischemic event or hepatic failure at the time of lactic acidosis^42, 43^. Metformin concentration in our study was determined by both preliminary experiment to exclude the toxic level to increase the release of lactic dehydrogenase in HPMCs (Supplementary Fig. 4). We also considered the peak plasma concentration reported in diabetic patients, 4–15 μM^45^. The concentration used in this study (10 μM) was within physiologic range in contrast to previous studies done in other cells, suggesting the possibility of safe use of metformin in PD patients with careful monitoring.

In this study, an improvement of peritoneal morphology with an induction of favorable oxidant/anti-oxidant balance in animal model of PD provided by metformin was not associated with a functional improvement of peritoneal transport (Supplementary Fig. 5). It is partly due to complicated interaction of multiple factors to determine peritoneal function in PD patients.

Our findings provide the first evidence that metformin ameliorate EMT and peritoneal fibrosis. Metformin decreased ROS generation with an enhanced anti-oxidant activity in peritoneum. Effect of metformin on peritoneal EMT seems to be mediated by both AMPK-dependent and -independent pathways. Our data suggests a potential therapeutic use of metformin or other AMPK agonists in prevention/treatment of peritoneal fibrosis.

## Methods

### Reagents

All chemicals and tissue culture plates were obtained from Sigma-Aldrich Co. (St. Louis, MO, USA) and Nunc Labware (Waltham, MA, USA), unless otherwise stated.

### Isolation and maintenance of HPMCs

Human peritoneal mesothelial cells (HPMCs) were isolated from human omentum and maintained using a modified method of previous publication^7^. Tissue collection was approved by the ethics committee of Ewha Womans University Mok-Dong Hospital and informed consent was obtained from each patient. Animal experiments were approved by the Animal Care and Use Committee of Kyungpook National University (KNU 2011-7) and conformed to the Guide for the Care and use of Laboratory Animals (NIH). All experiments were performed in accordance with relevant guidelines and regulations.

### Cell morphology and immunofluorescence analysis of HPMCs

Cell morphology was analyzed under an inverted phase-contrast microscope (Axiovert 200; Carl Zeiss, Oberkochen, Germany) and the images were obtained by digital camera (AxioCam HRC; Carl Zeiss). For immunofluorescence staining, cells were washed and fixed in 4% phosphate-buffered paraformaldehyde (25 minutes at 20 °C) and permeabilized with 1% Triton X-100 in PBS (15 minutes at 4 °C). After washing with PBS, the cells were treated with 5% BSA in PBS for 1 hour before incubation with primary antibodies specific for ZO-1 (Invitrogen, Carlsbad, CA, USA), α-SMA (Abcam, Cambridge, MA, USA) or β-catenin (Santa Cruz Biotechnology, Santa Cruz, CA, USA) in 5% BSA overnight at 4 °C. The cells were then washed with 0.2% Tween 20 in PBS before incubation with goat anti-mouse IgG-FITC-conjugated secondary antibody (Santa Cruz Biotechnology) for 1 hour at room temperature in the dark. The nucleus was counterstained with DAPI (4′,6-diamidino-2-phenylindole), and the cells were visualized under the Axiovert 200 fluorescence microscope with 10X10 and 20X10 NA objectives equipped with AxioCam HRC digital camera. Digital photographs were obtained with Axiovision 4.3 (Carl Zeiss) and merged images were obtained using Photoshop 7.0 (Adobe Systems, Toronto, Ontario, Canada).

### Preparation of protein extraction and western blotting

For the preparation of whole cell protein fraction, cells were collected with a cell scraper after washing cold PBS, and centrifuged at 15000 rpm for 5 min. After discarding supernatant, the cold RIPA buffer with protease inhibitor was added to cell pellet. Cells and RIPA buffer mixture were kept on ice for 15 min, and spun at 15000 rpm for 15 min at 4 °C.

The nuclear protein extraction was prepared using an NE-PER Nuclear Cytoplasmic Extraction Reagent kit (Pierce, Rockford, IL, USA) according to the manufacturer’s instruction. Briefly, the treated cells were washed twice with cold PBS and centrifuged at 500 *g* for 3 min. The cell pellet was suspended in 200 μl of cytoplasmic extraction reagent I by vortexing. The suspension was incubated on ice for 10 min followed by the addition of 11 μl of cytoplasmic extraction reagent II, vortexed for 5 s, incubated on ice for 1 min and centrifuged for 5 min at 16 000 *g*. The supernatant fraction (cytoplasmic extract) was transferred to a pre-chilled tube. The insoluble pellet fraction, which contains crude nuclei, was resuspended in 100 μl of nuclear extraction reagent by vortexing for 15 s and incubated on ice for 10 min, then centrifuged for 10 min at 16 000 *g*. The resulting supernatant, constituting the nuclear extract, was used for the subsequent experiments.

Protein lysates were run in SDS-PAGE for Western blot analysis. The blot was incubated overnight at 4 °C with primary antibodies directed to the following antigens: E-cadherin (BD Bioscience, Bedford, MA, USA), α-SMA, Collagen type I and SOD2 (Abcam), ZO-1 (Invitrogen), Snail, Phospho-GSK3β, GSK3β (Cell signaling, Danvers, MA, USA), fibronectin, β-catenin, Lamin B1, Phospho-Smad2, Phospho-Smad3, Smad2, Smad3, Phospho-ERK1/2, Phospho-p38, ERK1/2, p38, and β-actin (Santa Cruz Biotechnology). After washing the blot with PBS with Tween 20, blots were incubated with horse raddish peroxidase-conjugated secondary antibodies corresponding to each primary antibody followed by enhanced chemiluminescence detection (Santa Cruz Biotechnology). Positive immunoreactive bands were quantified by densitometry and compared with the expression of human β-actin.

### Extraction of total RNA and Real-time PCR

The levels of transcripts were determined by real time PCR (RT-PCR) on the ABI PRISM 7000 sequence detection system using SYBR Green I as a double-stranded DNA-specific dye (Applied Biosystems, Foster City, CA, USA). The PCR reaction was carried out in 1 µM of cDNA, 10 µL of SYBR Green PCR master mix, and 5 pM of sense and antisense primers of E-cadherin (Forward primer: ACCCCTGTTGGTGTCTTT, Reverse primer: TTCGGGCTTGTTGTCATTCT), α-SMA (Forward primer: GGGAATGGGACAAAAAG ACA, Reverse primer: CTTCAGGGGCAACACGAA), matrix metalloproteinase (MMP)-7 (Forward primer: AGTGGGAACAGGCTCAGGAC, Reverse primer: GCATCACCTC CAGAGTGTCG) for a final volume of 20 µL per reaction. The relative mRNA expression levels of the target genes in each sample were calculated using the comparative CT method. The CT value is the cycle number at which the fluorescence signal is greater than a defined threshold. At least three independent PCR procedures were performed to allow statistical analysis. The amount of PCR products was normalized with the house-keeping gene, β-actin, to determine the relative expression ratios for each mRNA in relation to the control group.

### TOP/FOP flash protocol

TOP/FOP plasmid and pCMV-RL were kindly provided by Professor Sangtaek Oh (Department of Bio and Fermentation Convergence Technology, Kookmin University, Seoul)^46^. Transient transfection was performed with Lipofectamine 3000 (Invitrogen, Carlsbad, CA) according to manufacturer’s instruction. In brief, cells were transfected with 0.1 μg of the luciferase reporter constructs (TOP flash or FOP flash) and 0.005 μg of Renilla reniformis gene for normalization. After 24 hour of transfection, TGFβ was added, and the cells were incubated for 24 hours. Luciferase assay were performed with the Dual Luciferase Assay Kit (Promega, Madison, WI) according to manufacturer’s instruction. Firefly luciferase activity was normalized to Renilla luciferase activity as the transfection control.

### ROS Generation by DCF-DA Fluorescence Measurement

HPMCs were incubated with 10 μM of 2′,7′-dichlorofluororescein diacetate (DCF-DA) (Invitrogen) for 30 minutes prior to an exposure to TGF-β1 (1 ng/mL) with or without metformin (1 µM). A serial fluorescence was measured using fluorescent plate reader at excitation 485 nm and emission 535 nm (Molecular device, Sunnyvale, CA, USA).

### Mitochondrial ROS Production

Mitochondria-associated ROS levels were measured by staining cells with 2.5 μM of MitoSOX^R^ red (Invitrogen), for 30 minutes at 37 °C. The cells were visualized under the Axiovert 200 fluorescence microscope up to 48 hours of TGF-β1 stimulation with or without metformin (1 µM). (Carl Ziss).

### Measurement of NADPH Oxidase (NOX) Activity

NOX activity was measured by a luminescence assay in a 50 mM phosphate buffer containing 1 mM EGTA, 150 mM sucrose, 5 μM lucigenin as the electron acceptor, and 100 μM NADPH as the donor with an addition of 100 μL of cell homogenate. Superoxide production was expressed as the rate of relative chemiluminescence units per milligram of protein. Protein content was measured using the Bio-Rad protein assay reagent (Bio-Rad Laboratories, Hercules, CA, USA).

### Gene Silencing of AMPK

To determine the effect of AMPK gene silencing on TGF-β1-induced EMT of HPMCs, we treated HPMCs with human AMPK siRNA obtained from Santa Cruz Biotechnology. The scrambled siRNA control was also selected from nontargeting siRNA pool (Santa Cruz Biotechnology). For transfection of siRNA, HPMCs were seeded into 6 wells for 24 hours at about 80% confluence, and then transfection of siRNA was performed using Lipofectamine RNAiMAX^TM^ (Invitrogen) according to the manufacturer’s protocol. After 24 hour of transfection media was changed, TGFβ was added, and the cells were incubated for 48 hours.

### Animal Model of Peritoneal Dialysis

Sprague-Dawley rats (250–300 g) were anesthetized with isoflurane. A 9-French silicone catheter (Access Technologies, Skokie, IL, USA) was inserted into the peritoneum through a small incision in the abdominal wall. The catheter was tunneled subcutaneously to an implanted port (Access Technologies). In one week after catheter implantation, daily infusion of dialysate was initiated. The dialysis solution of 25 ml (4.25% Dianeal^®^, pH 5.2; Baxter Healthcare, Deerfield, IL, USA) was infused twice daily (at 09:00 and 18:00 hr), 7 days per week, for 8 weeks. Control group (group C, n = 4) had a catheter inserted, but no fluid instilled. Dialysis group (group D, n = 4) was infused with a conventional dialysis solution (4.25% Dianeal^®^, pH 5.2; Baxter Healthcare). Rats in group D + M (n = 4) was infused with a dialysate and metformin (50 mg/kg/day).

### Morphologic and Immunofluorescence Analyses of Peritoneum

The parietal peritoneum of abdominal wall was fixed with 4% paraformaldehyde (pH 7.4) and embedded in paraffin. Parietal peritoneum sections of 2 μm thickness were stained with Masson’s trichrome. The thickness of the parietal peritoneum, including the mesothelium and submesothelial interstitium, was measured with an Aperio ScanScope CS Slide Scanner system (Aperio Technologies, Vista, CA, USA); whole-slide digital images were captured with X20 objective. For immunofluorescence microscopy, 3 μm tissue sections were incubated with primary antibodies against cytokeratin (Thermo Fisher Scientific, Waltham, MA, USA) or α-SMA (Abcam) at 4 °C overnight after antigen retrieval in boiling citrate buffer (pH 6.0) for 10 minutes. Sections were then incubated for 1 hours with fluorescein-conjugated secondary antibodies (Alexa Fluor 488 and Alexa Fluor 594; Molecular Probes, Eugene, OR, USA). The nuclei were counterstained with 4′,6-diamidino-2-phenylindole (DAPI; Molecular Probes), and slides were mounted with antifade mounting reagent (Molecular Probes). These sections were viewed under a confocal scanning laser microscope using LSM 5 EXCITER (Carl Zeiss).

### Immunohistochemistry of Peritoneal Oxidative Stress and Anti-oxidant Activity

Paraformaldehyde (4%)-fixed and paraffin-embedded tissue section (4 μm in thickness) were processed for immunohistochemical staining. Sections were de-paraffinized in xylene, rehydrated with graded ethanol, and washed with 0.1 M phosphate-buffered saline (PBS, pH 7.4). After microwave antigen retrival, sections were placed in a 0.3% H_2_O_2_ blocking solution for 15 minutes and then in 5% normal serum (Jackson Immuno Research Laboratories, West Grove, PA, USA) for 1 hour. Sections were incubated overnight, 4 °C with each of the following primary antibodies: SOD 2 (Abcam), NT (Santa Cruz Biotechnology), and 4-HNE (Abcam). Sections were then washed in 0.1 M PBS and incubated in corresponding biotinylated-secondary antibodies (Santa Cruz Biotechnology) for 1 hour. After washing, sections were incubated in ABC solution (Vector Vectastain Elite ABC kit, Vector, Burlingame, CA, USA) for 30 minutes, followed by a visualization with a diaminobenzidine (DAB) solution (DAB Peroxidase Substrate Kit, Vector). After counterstaining with Mayer’s hematoxylin, sections were dehydrated in graded ethanol, cleared in xylene, and coverslipped using Permount solution (Thermo Fisher Scientific). Sections were viewed and photographed with X20 objective under a light microscope (Zeiss Axioskop 2; Carl Zeiss)

### Measurement of oxidant/antioxidant status in HPMCs and peritoneal dialysate

To assess antioxidant status, the activities of glutathione reductase and SOD were measured in cell lysates or dialysate effluent using Glutathione Assay Kit (Cell Biolabs, San Diego, CA, USA) and Superoxide Dismutase Activity Assay (Cell Biolabs), respectively. Glutathione content was expresses as reduced/oxidized glutathione (GSH/GSSG). 8-hydroxy-2′-deoxyguanosine (8-OH-dG) content in dialysate were measured using Oxidative DNA Damage ELISA Kit (Cell Biolabs).

### Statistical Analysis

All data are presented as mean ± SD. Differences in the various parameters between groups were evaluated by ANOVA followed by correction for multiple comparison. Results were considered significant if the *P* value was ≤ 0.05.

## Electronic supplementary material

Supplementary Information

## References

[1] Krediet RT, Struijk DG (2013). Peritoneal changes in patients on long-term peritoneal dialysis. Nat Rev Nephrol.

[2] Williams JD (2002). Morphologic changes in the peritoneal membrane of patients with renal disease. J Am Soc Nephrol.

[3] Yanez-Mo M (2003). Peritoneal dialysis and epithelial-to-mesenchymal transition of mesothelial cells. N Engl J Med.

[4] Aroeira LS (2007). Epithelial to mesenchymal transition and peritoneal membrane failure in peritoneal dialysis patients: pathologic significance and potential therapeutic interventions. J Am Soc Nephrol.

[5] Selgas, R. *et al*. Epithelial-to-mesenchymal transition of the mesothelial cell–its role in the response of the peritoneum to dialysis. *Nephrol Dial Transplant* **21** Suppl 2 (2006). 10.1093/ndt/gfl183 16825254

[6] Yu MA (2009). HGF and BMP-7 ameliorate high glucose-induced epithelial-to-mesenchymal transition of peritoneal mesothelium. J Am Soc Nephrol.

[7] Shin HS, Ryu ES, Oh ES, Kang DH (2015). Endoplasmic reticulum stress as a novel target to ameliorate epithelial-to-mesenchymal transition and apoptosis of human peritoneal mesothelial cells. Lab Invest.

[8] Kriz W, Kaissling B, Le Hir M (2011). Epithelial-mesenchymal transition (EMT) in kidney fibrosis: fact or fantasy?. J Clin Invest.

[9] Taura, K., Iwaisako, K., Hatano, E. & Uemoto, S. Controversies over the Epithelial-to-Mesenchymal Transition in Liver Fibrosis. *J Clin Med* **5**, doi:10.3390/jcm5010009 (2016). 10.3390/jcm5010009 PMC4730134 26784242

[10] Shaw RJ (2013). Metformin trims fats to restore insulin sensitivity. Nat Med.

[11] Vazquez-Martin A (2010). Metformin regulates breast cancer stem cell ontogeny by transcriptional regulation of the epithelial-mesenchymal transition (EMT) status. Cell Cycle.

[12] Li L (2015). Metformin attenuates gefitinib-induced exacerbation of pulmonary fibrosis by inhibition of TGF-beta signaling pathway. Oncotarget.

[13] Yin M, Zhou J, Gorak EJ, Quddus F (2013). Metformin is associated with survival benefit in cancer patients with concurrent type 2 diabetes: a systematic review and meta-analysis. Oncologist.

[14] Cavaglieri RC, Day RT, Feliers D, Abboud HE (2015). Metformin prevents renal interstitial fibrosis in mice with unilateral ureteral obstruction. Mol Cell Endocrinol.

[15] Lee JH (2013). AMP-activated protein kinase inhibits TGF-beta-, angiotensin II-, aldosterone-, high glucose-, and albumin-induced epithelial-mesenchymal transition. Am J Physiol Renal Physiol.

[16] Li NS (2016). LKB1/AMPK inhibits TGF-beta1 production and the TGF-beta signaling pathway in breast cancer cells. Tumour Biol.

[17] Lim JY, Oh MA, Kim WH, Sohn HY, Park SI (2012). AMP-activated protein kinase inhibits TGF-beta-induced fibrogenic responses of hepatic stellate cells by targeting transcriptional coactivator p300. J Cell Physiol.

[18] Xiao H (2010). Metformin attenuates cardiac fibrosis by inhibiting the TGFbeta1-Smad3 signalling pathway. Cardiovasc Res.

[19] Lan A, Qi Y, Du J (2014). Akt2 mediates TGF-beta1-induced epithelial to mesenchymal transition by deactivating GSK3beta/snail signaling pathway in renal tubular epithelial cells. Cell Physiol Biochem.

[20] Caraci F (2008). TGF-beta1 targets the GSK-3beta/beta-catenin pathway via ERK activation in the transition of human lung fibroblasts into myofibroblasts. Pharmacol Res.

[21] Ji Q (2015). Resveratrol suppresses epithelial-to-mesenchymal transition in colorectal cancer through TGF-beta1/Smads signaling pathway mediated Snail/E-cadherin expression. BMC Cancer.

[22] Liu Y (2015). Transition of mesothelial cell to fibroblast in peritoneal dialysis: EMT, stem cell or bystander?. Perit Dial Int.

[23] Jang YH (2013). Effects of dexamethasone on the TGF-beta1-induced epithelial-to-mesenchymal transition in human peritoneal mesothelial cells. Lab Invest.

[24] Yu M (2015). Effect of aldosterone on epithelial-to-mesenchymal transition of human peritoneal mesothelial cells. Kidney Res Clin Pract.

[25] Loureiro J (2013). Tamoxifen ameliorates peritoneal membrane damage by blocking mesothelial to mesenchymal transition in peritoneal dialysis. PLoS One.

[26] Prasad CP (2009). Expression analysis of E-cadherin, Slug and GSK3beta in invasive ductal carcinoma of breast. BMC Cancer.

[27] Zhao Z (2014). Metformin inhibits the IL-6-induced epithelial-mesenchymal transition and lung adenocarcinoma growth and metastasis. PLoS One.

[28] Qu C (2014). Metformin reverses multidrug resistance and epithelial-mesenchymal transition (EMT) via activating AMP-activated protein kinase (AMPK) in human breast cancer cells. Mol Cell Biochem.

[29] Li L (2014). Metformin sensitizes EGFR-TKI-resistant human lung cancer cells *in vitro* and *in vivo* through inhibition of IL-6 signaling and EMT reversal. Clin Cancer Res.

[30] Zhang R (2015). Inhibitory effects of metformin at low concentration on epithelial-mesenchymal transition of CD44(+)CD117(+) ovarian cancer stem cells. Stem Cell Res Ther.

[31] Zhang J (2014). Metformin inhibits epithelial-mesenchymal transition in prostate cancer cells: involvement of the tumor suppressor miR30a and its target gene SOX4. Biochem Biophys Res Commun.

[32] Lee HB, Yu MR, Song JS, Ha H (2004). Reactive oxygen species amplify protein kinase C signaling in high glucose-induced fibronectin expression by human peritoneal mesothelial cells. Kidney Int.

[33] Cahova M (2015). Metformin prevents ischemia reperfusion-induced oxidative stress in the fatty liver by attenuation of reactive oxygen species formation. Am J Physiol Gastrointest Liver Physiol.

[34] Morales AI (2010). Metformin prevents experimental gentamicin-induced nephropathy by a mitochondria-dependent pathway. Kidney Int.

[35] Bhatt MP, Lim YC, Kim YM, Ha KS (2013). C-peptide activates AMPKalpha and prevents ROS-mediated mitochondrial fission and endothelial apoptosis in diabetes. Diabetes.

[36] Cai L (2015). AMPK dependent protective effects of metformin on tumor necrosis factor-induced apoptotic liver injury. Biochem Biophys Res Commun.

[37] Liem DA, Honda HM, Zhang J, Woo D, Ping P (2007). Past and present course of cardioprotection against ischemia-reperfusion injury. Journal of applied physiology.

[38] Hawley SA, Gadalla AE, Olsen GS, Hardie DG (2002). The antidiabetic drug metformin activates the AMP-activated protein kinase cascade via an adenine nucleotide-independent mechanism. Diabetes.

[39] Lee SY (2015). Metformin Ameliorates Inflammatory Bowel Disease by Suppression of the STAT3 Signaling Pathway and Regulation of the between Th17/Treg Balance. PLoS One.

[40] Soraya H (2012). Anti-angiogenic Effects of Metformin, an AMPK Activator, on Human Umbilical Vein Endothelial Cells and on Granulation Tissue in Rat. Iran J Basic Med Sci.

[41] Thakur S (2015). Activation of AMP-activated protein kinase prevents TGF-beta1-induced epithelial-mesenchymal transition and myofibroblast activation. Am J Pathol.

[42] DeFronzo R, Fleming GA, Chen K, Bicsak TA (2016). Metformin-associated lactic acidosis: Current perspectives on causes and risk. Metabolism.

[43] Salpeter, S. R., Greyber, E., Pasternak, G. A. & Salpeter, E. E. Risk of fatal and nonfatal lactic acidosis with metformin use in type 2 diabetes mellitus. *Cochrane Database Syst Rev* CD002967 (2010). 10.1002/14651858.CD002967.pub3 20091535

[44] Al-Hwiesh AK (2014). Metformin in peritoneal dialysis: a pilot experience. Perit Dial Int.

[45] Graham GG (2011). Clinical pharmacokinetics of metformin. Clinical pharmacokinetics.

[46] Gwak J (2012). Small molecule-based disruption of the Axin/beta-catenin protein complex regulates mesenchymal stem cell differentiation. Cell research.

