# Supplementary material for: Metformin ameliorates the Phenotype Transition of Peritoneal Mesothelial Cells and Peritoneal Fibrosis via a modulation of Oxidative Stress
Source: Sci Rep. 2017 Jul 18;7:5690. doi: 10.1038/s41598-017-05836-6 (PMC5515908; doi:10.1038/s41598-017-05836-6)

**Supplementary Data**

**Metformin inhibits Phenotype Transition of Peritoneal Mesothelial Cells and Peritoneal Fibrosis**

**via an Amelioration of Oxidative Stress**

Hyun-Soo Shin, Ph.D.**1**, Jiyeon Ko, M.S.1, Dal-Ah Kim, Ph.D.1, Eun-Sun Ryu, M.S.**1**, Hye-Myung Ryu, M.D, Ph.D.2, Sun-Hee Park, M.D, Ph.D.2, Yong-Lim Kim, M.D, Ph.D.2, Eok-Soo Oh, Ph.D.3, and Duk-Hee Kang, M.D, Ph.D.**1**

1Division of Nephrology, Department of Internal Medicine, Ewha Womans University School of Medicine, Ewha Medical Research Center, Seoul; 2Division of Nephrology, Department of Internal Medicine, Kyung-Pook National University School of Medicine, Dae-gu and 3Department of Life Sciences, Division of Life and Pharmaceutical Sciences, Ewha Womans University, Seoul, Republic of Korea

**Supplemental Methods**

**Effect of metformin on cell proliferation and cytotoxicity**

To determine the concentration of metformin for the experiments using HPMCs, we first examined the effect of metformin on cell proliferation and cytotoxicity. A 3-(4,5-dimethylthiazol-2-yl)-5-(3-carboxymethoxyphenyl)-2-(4-sulfophenyl)-2H-tetrazolium (MTS) uptake assay was used for assessing cell proliferation (Promega, Madison, WI). The cells were treated with metformin (0 to 100 μM) for 48 hours. After the addition of 20 μl of MTS reagent and incubation at 37°C for 4 hours, proliferation of HPMCs was measured by reading the absorbance at 490 nm (Dynex Revelation, Dynex Ltd., Billingshurst, UK). Cytotoxicity of metformin in HPMCs was determined by the amount of lactate dehydrogenase (LDH) leaking into the medium after 6-48 hours of treatment using the LDH cytotoxicity detection kit (Roche, Mannheim, Germany). Each data series was analyzed in triplicate.

**Transfection of siRNA**

To determine the effect of Smad2/3 gene silencing on TGF-β1-induced EMT of HPMCs, we treated HPMCs with human Smad2 and Smad3 siRNA obtained from Thermo Fisher Scientific (Waltham, MA, USA). The following siRNAs were used: Smad2, ON-TARGETplus SMARTpool human Smad2 (4087); Smad3 siRNA, ON-TARGETplus SMARTpool human Smad3 (4088). The scrambled siRNA control is a nontargeting siRNA pool from Thermo Fisher Scientific. For transfection of siRNA, HPMCs were seeded into 6 wells for 24 hours at about 80% confluence, and then transfection of siRNA was performed using Lipofectamine RNAiMAXTM (Invitrogen) according to the manufacturer's protocol.

**Peritoneal equilibrium test (PET)**

Peritoneal membrane function was assessed at baseline and 8 weeks after initiating dialysis using the 4 h PET employing 4.25% glucose solution (Dianeal®). Dialysate samples were taken at 0 and 4 h. Dialysate glucose concentrations were determined by the glucose oxidase method. All animals were weighed at 0 and 8 weeks. The peritoneal membrane transport rate was assessed by the ratio of dialysate glucose at 4 h dwell time to dialysate glucose at 0 h (D/D0 glucose). Dialysate to plasma ratios were also calculated for urea (D/P urea).

**Supplemental Figures Captions**

**Supplementary Figure S1. Role of Smad2/3 and p38/erk MAPKinase Activation on TGFβ1-induced EMT of HPMCs.**

TGF-β1 induced the phosphorylation of Smad2/3 from 30 minutes (A), and activated p38 and ERK1/2 MAPK pathways from 3 hours of stimulation (B). Gene silencing of Smad2/3 by siRNA ameliorated TGF-β1-induced EMT (C). Inhibition of p38 MAPK (SB203589, 10 μM) or ERK1/2 MAPK (PD98059, 10 μM) also alleviated EMT in HPMCs exposed to TGF-β1. N=6. *p<0.05 vs. others, #p<0.05 vs. TGF-β1.

**Supplementary Figure S2. Activation of AMPK by Metformin and AICAR in HPMCs.**

AICAR (10 μM) activated AMPK of HPMCs from 15 minutes. However, metformin (10 μM) induced the phosphorylation of AMPK from 24 hours in HPMC (A). Compound C (20 μg/mL) inhibited AMPK activity induced by AICAR or metformin at 48 hours (B). N=4. *p<0.05 vs. others.

**Supplementary Figure S3. Effect of Anti-oxidants on TGFβ1-induced EMT of HPMCs.**

TGF-β1-induced alteration in the expression of E-cadherin and α-SMA significantly ameliorated by treatment with anti-oxidants, N-acetyl cystein (NAC, 5 mM), Apocynin (10 μM) or MitoQ (1 μM). N=5, *p<0.05 vs. others, #p<0.05 vs. TGF-β1.

**Supplementary Figure S4. Effect of Metformin on Proliferation and Cytotoxicity of HPMCs.**

N=5, *p<0.05 vs. others.

**Supplementary Figure S5. Body Weight and Peritoneal Function in Animal Model of Peritoneal Dialysis.**

A Peritoneal equilibrium test (PET) was performed at baseline and 8 weeks after initiating dialysis. At 8 weeks of peritoneal dialysis, body weight gain was lower in group D+M compared to the rats in groups C and D (A). At baseline and 8 weeks of peritoneal dialysis, the ratio of dialysate glucose at 4 h dwell time to dialysate glucose at 0 h (D/D0 glucose) was significantly decreased at 8 weeks, but no statistical difference was observed among 3 groups (B). Dialysate to plasma urea ratio (D/P urea) at 8 weeks of peritoneal dialysis tends to be lower in D+M group compared to C or D, however it did not reach a statistical significance. *p<0.05 vs. C and D at 8 weeks, #p<0.05 vs. corresponding groups at baseline.


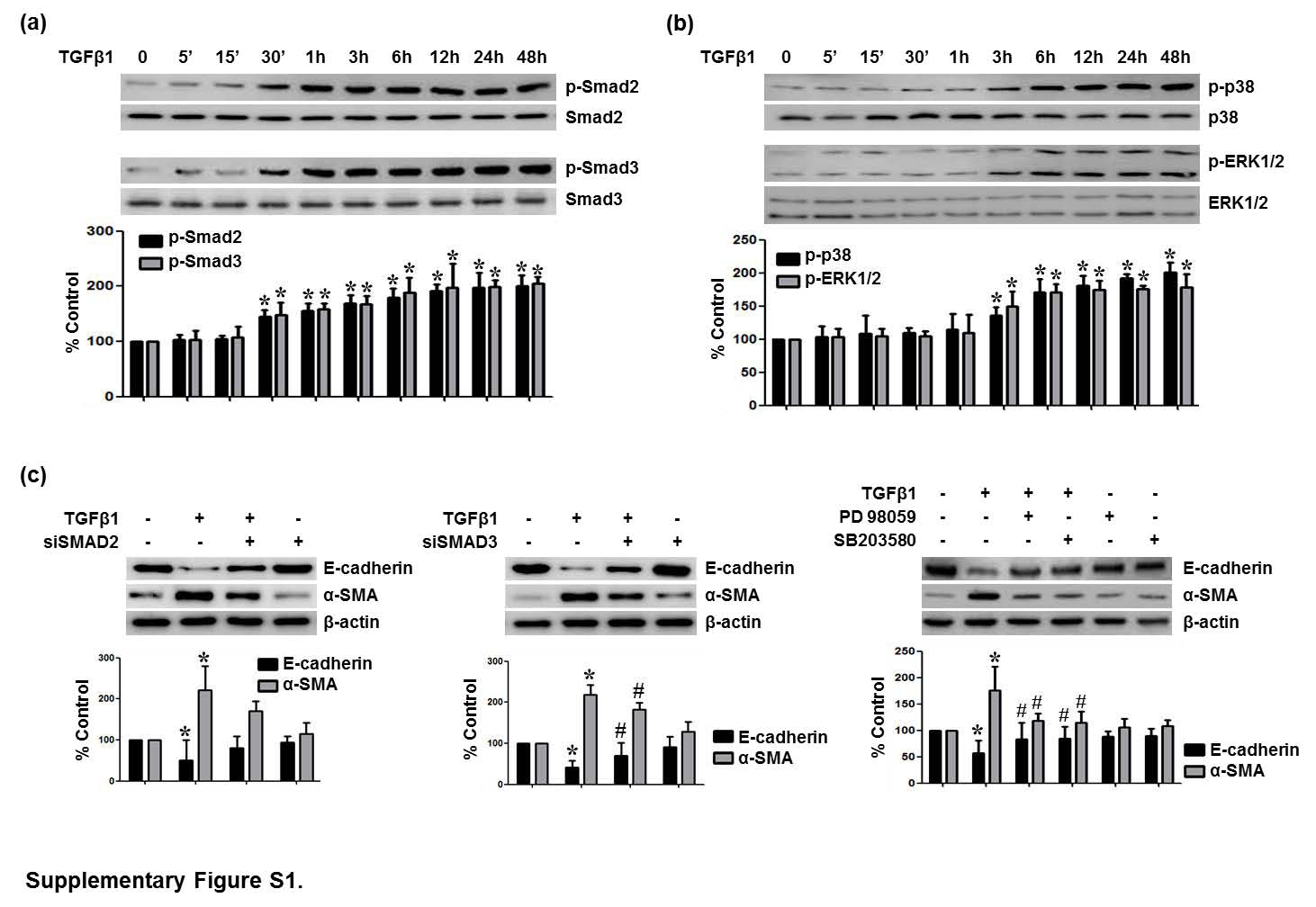


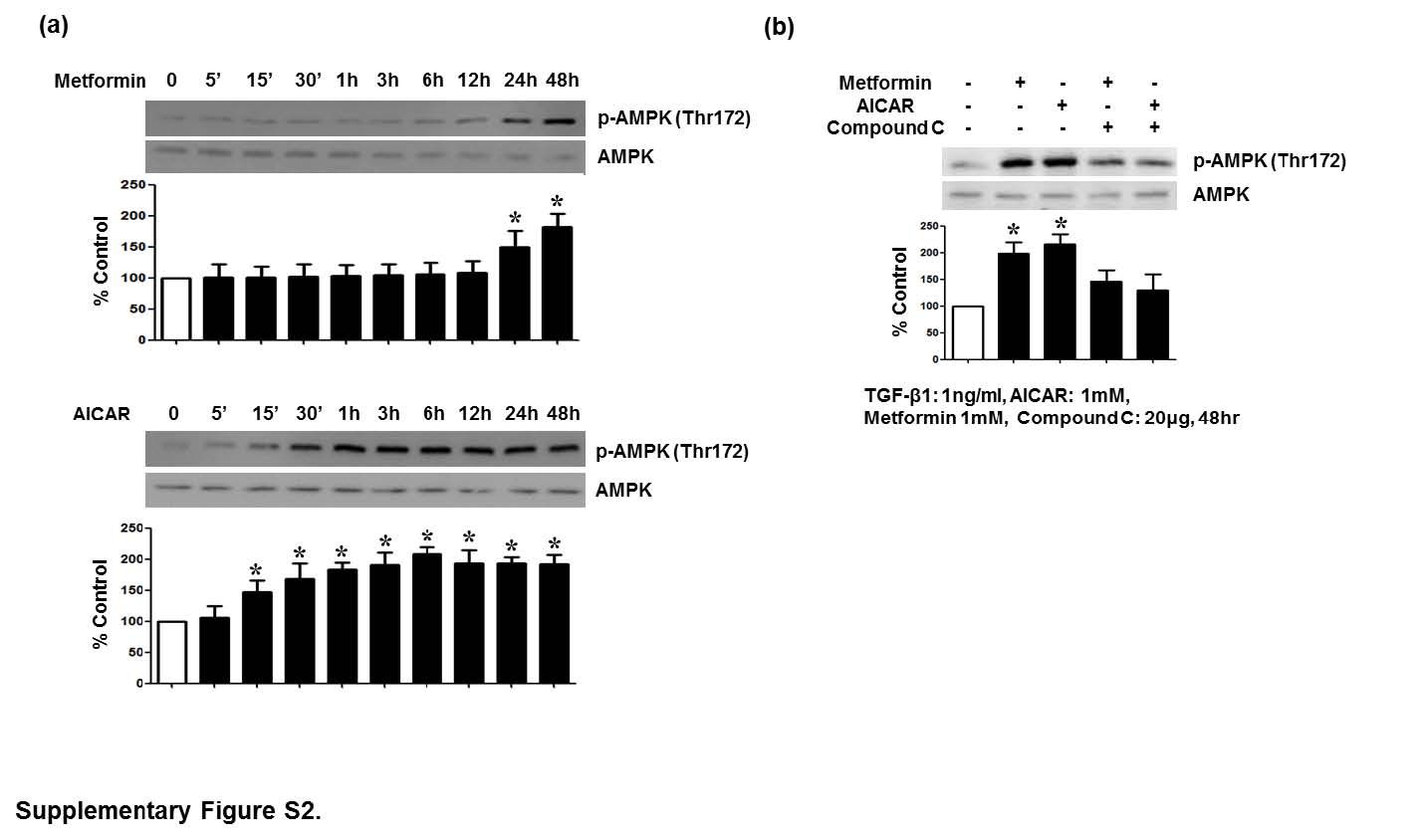


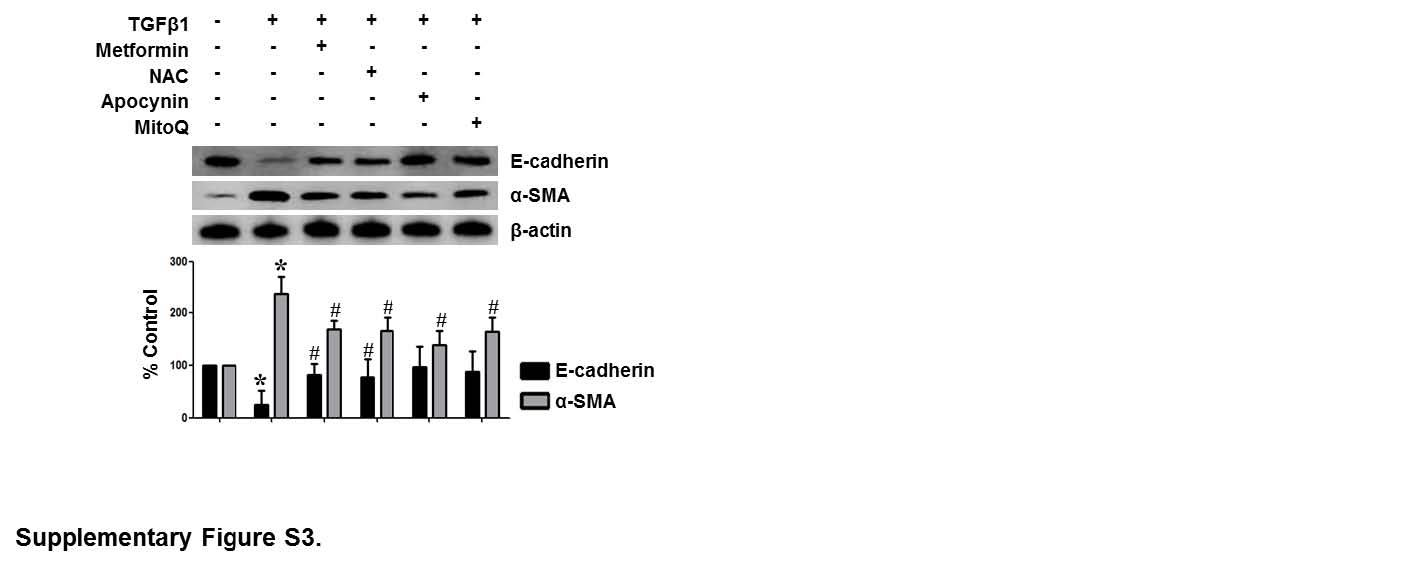


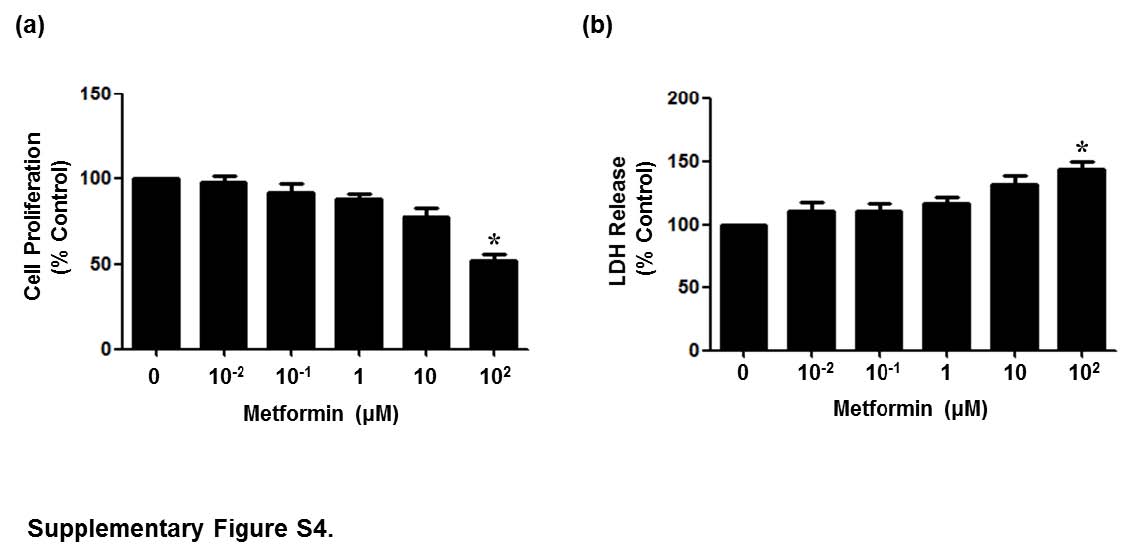


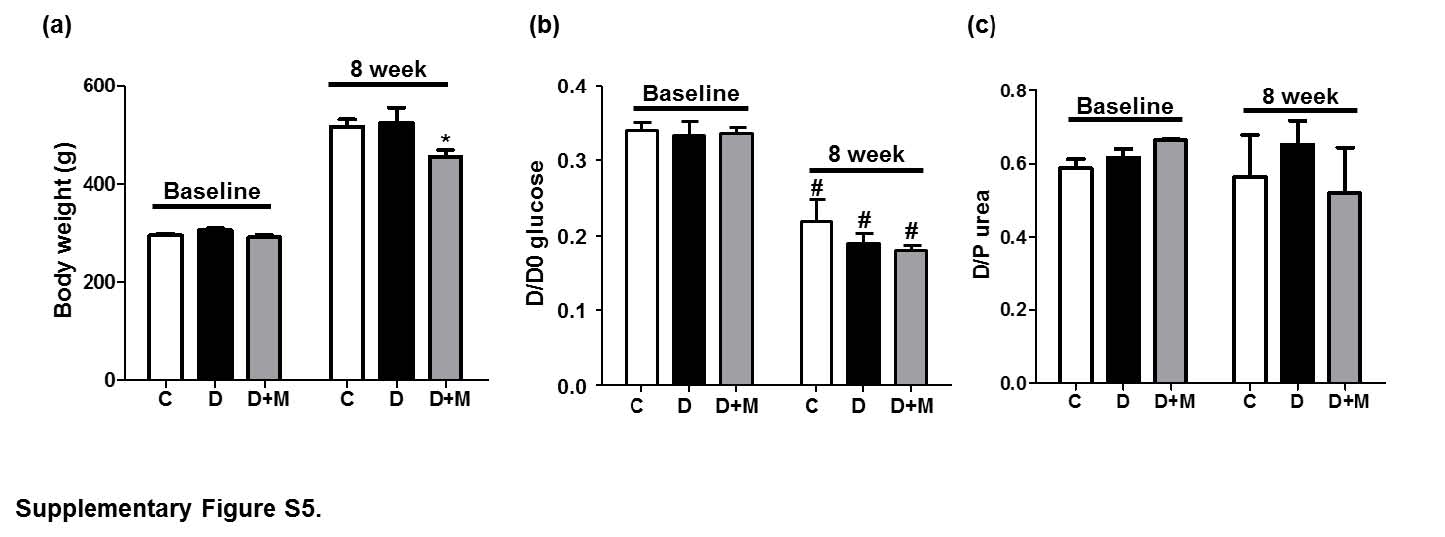

Supplement: Supplementary file 1 — Supplementary Information [file 41598_2017_5836_MOESM1_ESM.doc]
